# Using EEG to challenge ASD heterogeneity: Stratification of brain functional connectivity reveals clinically meaningful subgroups of ASD

**DOI:** 10.1192/j.eurpsy.2025.730

**Published:** 2025-08-26

**Authors:** B. Rodriguez-Herreros, A. Mheich, S. Yassine, J. M. A. Osório, S. Richetin, V. Junod, L. Mendes, K. Gschwend, V. Aeschbach, L. Arnold, D. Romascano, P. Yu, M. Jequier Gygax, A. M. Maillard, M. Hassan, N. Chabane

**Affiliations:** 1 STSA, CHUV, Lausanne, Switzerland; 2 Université de Rennes; 3 MINDIG, Rennes, France; 4 Nuffield Department of Clinical Neurosciences, Oxford University, Oxford, United Kingdom; 5 Département femme-mère-enfant, CHUV, Lausanne, Switzerland; 6 School of Science and Engineering, Reykjavik University, Reykjavik, Iceland

## Abstract

**Introduction:**

Heterogeneity in both the etiology and the phenotypic presentation of autism spectrum disorder (ASD) poses a major challenge to clinical and translational research. Attempts to stratify individuals with ASD have been primarily based on behavioral criteria, but clinical subtyping is blind to the underlying neurobiological mechanisms and has limited predictive value of the forthcoming developmental path. Yet, it is still unclear whether and how atypical brain functional connectivity can account for individual differences across ASD-related symptomatology and behaviors.

**Objectives:**

The goal of the study was to identify clinically meaningful subgroups of young children with ASD based on distinctive patterns of brain functional connectivity, to better understand the neural substrates underlying ASD heterogeneity.

**Methods:**

We combined resting-state EEG data from 4 independent datasets on 541 children with ASD aged 2-12 years to estimate and stratify brain functional connectivity measures. We performed an unsupervised clustering analysis of the cortical network properties, using data-driven similarity network fusion and source-based spectral analysis. We then compared the clinical profile of the identified clusters to define symptom-linked connectivity dimensions.

**Results:**

We identified four subgroups of ASD children with distinct cortical network properties, mainly mapped in the fronto-parietal and precentral cortices for the alpha band, and in the middle temporal cortex for beta band. These four clustered dimensions of functional connectivity were associated to distinctive different clinical symptom profiles, specially with respect to cognitive level, adaptive behavior and motricity.

**Image 1:**

**Figure:**
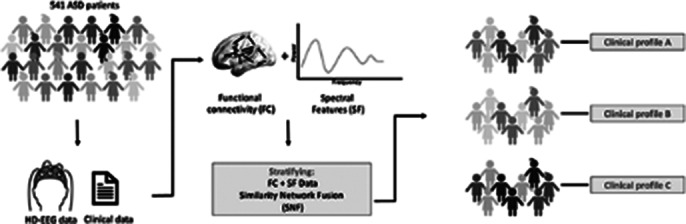

**Conclusions:**

Our findings shed light on atypical brain network topology conferring risk for specific phenotypic manifestations of ASD, which may implicate unique underlying neurobiological mechanisms. Cross-validation stability hints at a solid stratification model to challenge ASD heterogeneity. Collectively, the stratification of well-defined neural signatures that give rise to the clinical heterogeneity of ASD has the potential to provide more accurate prognosis and help to select the optimal strategy for therapeutic intervention.

**Disclosure of Interest:**

None Declared

